# Enhanced Solubilization of Fluoranthene by Hydroxypropyl β-Cyclodextrin Oligomer for Bioremediation

**DOI:** 10.3390/polym10020111

**Published:** 2018-01-24

**Authors:** Kyeong Hui Park, Jae Min Choi, Eunae Cho, Seunho Jung

**Affiliations:** 1 Department of Systems Biotechnology, Microbial Carbohydrate Resource Bank (MCRB), Center for Biotechnology Research in UBITA (CBRU), Konkuk University, Seoul 05029, Korea; kyeonghee17@naver.com; 2 Center for Biotechnology Research in UBITA (CBRU), Institute for Ubiquitous Information Technology and Applications (UBITA), Konkuk University, Seoul 05029, Korea; shoyalove@naver.com (J.M.C.); echo@konkuk.ac.kr (E.C.)

**Keywords:** hydroxypropyl β-cyclodextrin, fluoranthene, polycyclic aromatic hydrocarbons

## Abstract

Fluoranthene (FT) is a polycyclic aromatic hydrocarbon (PAH), consisting of naphthalene and benzene rings connected by a five-member ring. It is widespread in the environment. The hydrophobicity of FT limits its availability for biological uptake and degradation. In this study, hydroxypropyl β-cyclodextrin oligomers (HP-β-CD-ol) were synthesized with epichlorohydrin (EP), while the solubility enhancement of FT by HP-β-CD-ol was investigated in water. The synthesized HP-β-CD-ol was characterized by MALDI-TOF mass spectrometry (MS), ^1^H NMR, and ^13^C NMR spectroscopy. The solubility of FT increased 178-fold due to the complex formation with HP-β-CD oligomers. The inclusion complexes of FT/HP-β-CD-ol were analyzed using Fourier-Transform Infrared (FT-IR), Differential Scanning Calorimetry (DSC), Scanning Electron Microscope (SEM), and Nuclear Overhauser Effect Spectroscopy Nuclear magnetic resonance (NOESY NMR) spectroscopy. On the basis of these results, HP-β-CD-ol is recommended as a potential solubilizer for the development of PAH removal systems.

## 1. Introduction

Polycyclic aromatic hydrocarbons (PAHs) have caused considerable concern as environmental organic pollutants that are highly carcinogenic or mutagenic. They are prevalent in air, soil, and water [1]. PAHs remain in the environment for a long time due to their high persistence, without degradation. PAHs are hydrophobic organic compounds that are composed of two or more aromatic rings. They are found in coal and tar, and are generated by the incomplete combustion of organic materials such as fuel and biomass [2]. PAHs have chemical stability, extremely low water solubility and low bioavailability [3]. Therefore, there is a limit to the removal of PAHs from soil and waste water. Various solubility enhancing agents, such as surfactants [4] and co-solvents [5], have been used to improve the desorption efficiency of PAH from soil, which have enhanced the mobility and bioavailability of PAHs in the aqueous phase [6]. However, it can be difficult to remove, while being toxic to the resident microbial populations and humans [6].

Fluoranthene (FT) is composed of four aromatic rings and is a member of the most abundant high-molecular-weight PAHs of various xenobiotics. FT has been identified in the atmosphere, on some surfaces, in drinking and waste-water, and charcoal grilled foods [7]. FT can be absorbed through the skin following dermal exposure [8]. It can also be absorbed into the gastrointestinal tract and lungs [9]. FT has been classified as a Group 3 carcinogen by the International Cancer Research Agency [10], since it is a genotoxic [11] and mutagenic carcinogen [12]. However, it has high hydrophobicity and is not readily accessible for biological absorption or degradation, thus increasing its persistence in the environment.

β-cyclodextrin (β-CD) has been proposed as a substitute to enhance the water dispersion of hydrophobic compounds through inclusion complexes. β-CD is a cyclic heptasaccharide, containing α-(1,4) linked glucose units, and is produced by bacterial enzymes from starch. β-CD has unique properties—The inner cavity is hydrophobic, while the outer is hydrophilic [13]. Based on the structure, they are capable of forming inclusion complexes with hydrophobic compounds, which non-covalently interact with the hydrophobic interior cavity of β-CD [13,14]. Furthermore, the solubility and bioavailability of hydrophobic materials can be improved through inclusion complexes with β-CD [15,16]. Chemical modification techniques have been introduced to extend the application of natural β-CD. Their functional hydroxyl groups are modified with specific substituents by chemical derivatization [17]. However, the oligomer types of β-CD or β-CD derivatives have not been significantly studied so far, with respect to the effective complexation with target molecules. In particular, hydroxypropyl β-cyclodextrin HP-β-CD is biocompatible, with better aqueous solubility (>60%) than the original β-CD (1.8%) [18,19]. In this study, HP β-CD oligomer was synthesized through a 1-step reaction using epichlorohydrin. Subsequently, its effective complexation with FT was studied.

In this study, we proposed a chemically modified epichlorohydrin cross-linked hydroxypropyl-β-cyclodextrin oligomer (HP-β-CD-ol) as a novel solubilizer for its ability to enhance the aqueous solubility and bioremediation of FT. The synthesized HP-β-CD-ol was analyzed by MALDI-TOF MS, ^1^H NMR, and ^13^C NMR spectroscopy. The improved solubility of FT, using the modified β-CD oligomer thus synthesized, was compared with β-CD derivatives. The apparent constant and solubility efficiency of FT/HP-β-CD-ol were determined using the phase solubility method. The formation of the inclusion complex was characterized by FT-IR, DSC and SEM. NOESY was studied to predict the three-dimensional structure of the inclusion complex between HP-β-CD-ol and FT.

## 2. Materials and Methods

### 2.1. Chemicals

FT was purchased from Wacker-Chemie (Lyon, France), while HP-β-CD (MW 1540) was purchased from Sigma-Aldrich chemical Co. (St. Louis, MO, USA). HPLC grade of Epichlorohydrin (EP) and methanol (MeOH) were obtained from Fisher Scientific (Illkirch, France). Deionized water was produced with a Milli-Q system from Millipore (Saint-Quentin-en-Yvelines, France). Other chemicals used in the study were of analytical grade.

### 2.2. Synthesis of HP-β-CD-ol

The EP cross-linked HP-β-CD-ol was synthesized with a slight modification of the previously reported method [20]. Firstly, the HP-β-CD was dissolved in NaOH solution (5%, *w/w*) completely by magnetically stirring at 25 °C for 24 h. EP was added dropwise to the HP-β-CD solution. The molar ratio of HP-β-CD to EP was 1:10. The mixture was heated to 60 °C and stirred at 600 rpm for 5 h. The reaction was terminated by neutralization with 3 N HCl. For the removal of HP-β-CD monomer derivatives, the reaction mixture was applied on the Bio-Gel P4 column. The separated HP-β-CD-ol was identified by MALDI-TOF mass spectrometry (Applied Biosystems, Foster city, CA, USA) and NMR spectroscopy (Bruker GmbH, Karlsruhe, Germany).

### 2.3. HP-β-CD-ol Characterization

#### 2.3.1. MALDI-TOF MS

The sample solution contained 1 μL of HP-β-CD-ol and 1 μL of 2,5-dihydroxybenzoic acid (DHB) solution (10 mg/mL DHB/H_2_O solution) as a matrix was deposited on the 96 well plate using the dried droplet method. The dried sample was analyzed using the AB SCIEX MALDI TOF-TOF 5800 System (Applied Biosystems, Foster city, CA, USA). All mass spectra were acquired in positive linear mode with an accelerating voltage of 20 kV in the reflector mode. In addition, each spectrum is an Average of 500 laser shots.

#### 2.3.2. ^1^H NMR and ^13^C NMR Spectroscopy

^1^H NMR and ^13^C NMR spectroscopy were carried out using a Bruker Avance 500 MHz spectrometer (Bruker GmbH, Karlsruhe, Germany) at room temperature by dissolving 5 mM of the samples in 600 μL of deuterated water (D_2_O, 99.96%).

### 2.4. Solubility Enhancement Test

β-CD, randomly dimethyl β-cyclodextrin (RM-β-CD), sulfobutyl ether β-cyclodextrin (SBE-β-CD), HP-β-CD, and HP-β-CD-ol were used to test the solubility of FT in water. A fixed amount (200 µg) of FT was added to 1 mL aqueous solution of β-CD derivatives and HP-β-CD-ol (1 mM). Subsequently, the mixture was stirred at 400 rpm for 24 h at 25 °C in the dark. The samples were filtered through a PTFE 0.20 μm filter (Advantec MFS, CA, USA) and measured with a UV-Vis spectrophotometer (UV 2450, Shimadzu Corporation, Kyoto, Japan).

### 2.5. Phase Solubility Analysis

Phase solubility study was performed using the previously reported procedure by Higuchi and Conners [21]. The aqueous solution of HP-β-CD and HP-β-CD-ol was prepared at a concentration of 0, 0.02, 0.05, 0.09, 0.19, 0.38, 0.75 and 1.5 mM. Excess FT (200 µg) was added to 1 mL of various concentrations of HP-β-CD and HP-β-CD-ol solution in capped 5 mL vials. Subsequently, the mixtures were magnetically stirred at 400 rpm for 24 h at 25 °C in the dark. After equilibrium was achieved, the samples were filtered through a PTFE 0.20 μm filter (Advantec MFS, CA, USA). Concentrations of dissolved FT were measured at 276 nm using a UV-Vis spectrophotometer. The apparent binding constants (*K*_m:n_) of the FT/HP-β-CD derivative complexes were calculated based on the phase solubility diagrams. The binding constants (*K*_m:n_) for the formation of the inclusion complexes can be expressed as:

(1) $$K_{1:1}=\frac{slope1}{S_{o}\left(1-slope1\right)}$$

(2) $$K_{2:1}=\frac{slope2}{K_{1:1}S_{o}}$$

(3) $${\left[S\right]}_{t}=S_{o}+K_{1:1}S_{o}\left[Host\right]+K_{1:1}K_{2:1}S_{o}{\left[Host\right]}^{2}$$

where *S*_0_ is the equilibrium solubility of FT in the absence of HP-β-CD-ol. The solubilization efficiency (SE) was obtained using the ratio between the concentration of solubilized FT and the total concentration of FT added:

(4) $$\mathrm{SE}=\;\frac{S_{e}}{S_{0}}$$

where *S_e_* is the concentration of FT solubilized by the solubilizer and *S*_0_ is concentration of FT in absence of solubilizer.

### 2.6. Method of Sample Preparation

#### 2.6.1. Preparation of the Physical Mixture (PM)

The PM was prepared by simple homogenization method. FT powder and lyophilized HP-β-CD-ol powder were grinded in a molar ratio of 1:1 using a mortar and pestle.

#### 2.6.2. Preparation of the Inclusion Complex (IC)

The IC of FT with HP-β-CD-ol was prepared by the freeze-drying method. Equimolar concentrations of FT and HP-β-CD-ol were added in distilled water, subsequently being stirred at 25 °C in the dark. After 24 h, the samples were filtered to remove uncomplexed FT. The filtered solution was freeze-dried to obtained solid complexes for characterization.

### 2.7. Inclusion Complex Characterization

#### 2.7.1. Fourier-Transform Infrared (FT-IR)

FT-IR spectra of FT, HP-β-CD-ol, FT/HP-β-CD-ol PM, and FT/HP-β-CD-ol IC were obtained using Nicolet 6700 (Thermo Scientific, Waltham, MA, USA) with attenuated total reflection (ATR) technique. The scanning range was 650–4000 cm^−1^, while the resolution was set to 8 cm^−1^.

#### 2.7.2. Differential Scanning Calorimetry (DSC)

DSC analyses of FT, HP-β-CD-ol, PM, and IC were recorded using the Discovery DSC (TA Instrument, New Castle, DE, USA). Samples were prepared in aluminum pans and scanned in the temperature range of 50–300 °C. The scanning rate was 10 °C/min under a nitrogen flow of 100 mL/min.

#### 2.7.3. Scanning Electron Microscope (SEM)

The morphology of FT, HP-β-CD-ol, PM, and IC were obtained by Hitachi S-4700 (Hitachi High-Technologies Corporation, Tokyo, Japan). The samples were fixed on a brass stub using double-sided adhesive carbon tape. They were coated with a thin layer of gold for 30 s to become electrically conductive.

#### 2.7.4. Nuclear Overhauser Effect Spectroscopy (NOESY)

The 2D NMR experiment of FT/HP-β-CD-ol IC was performed with a Bruker Avance 500 MHz spectrometer (Bruker GmbH, Karlsruhe, Germany) in D_2_O at 25 °C.

## 3. Results and Discussion

### 3.1. Synthesis of Hydroxypropyl β-Cyclodextrin Oligomer (HP-β-CD-ol)

**Figure 1:**
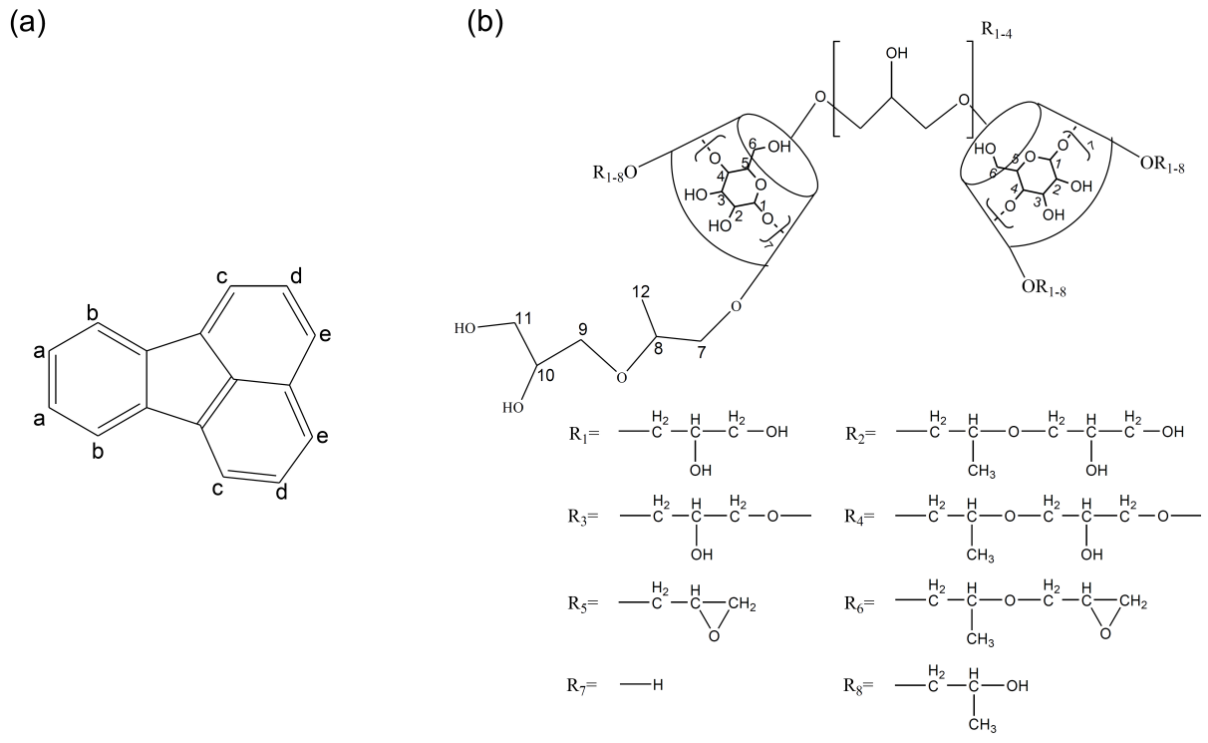
Chemical structure of (**a**) FT and (**b**) HP-β-CD-ol.

**Figure 2:**
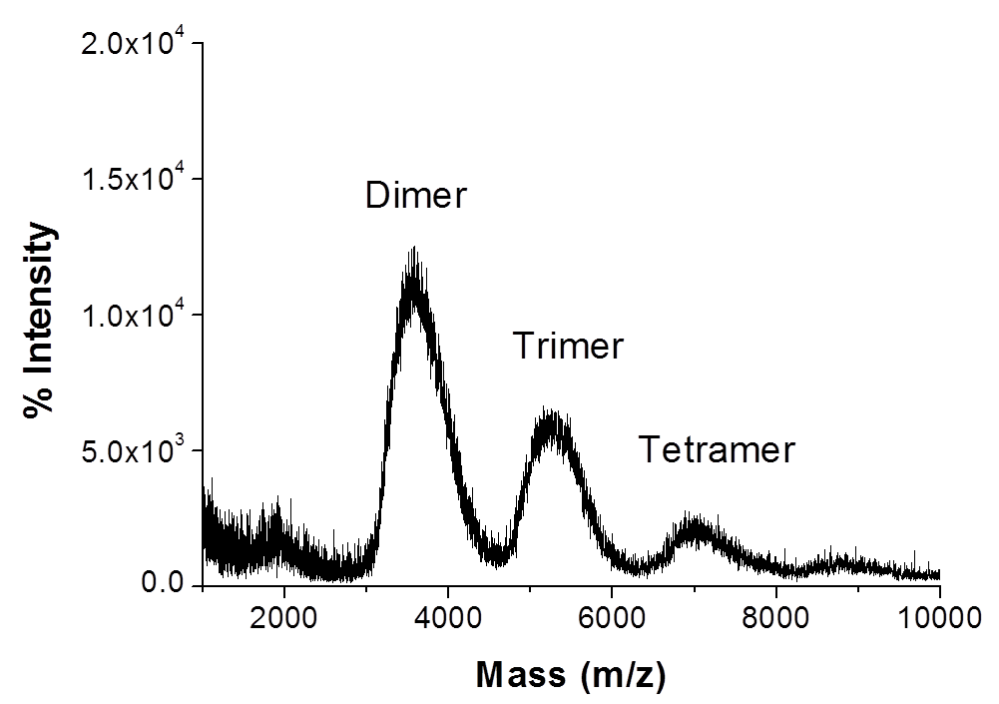
MALDI-TOF mass spectrum of HP-β-CD-ol.

**Figure 3:**
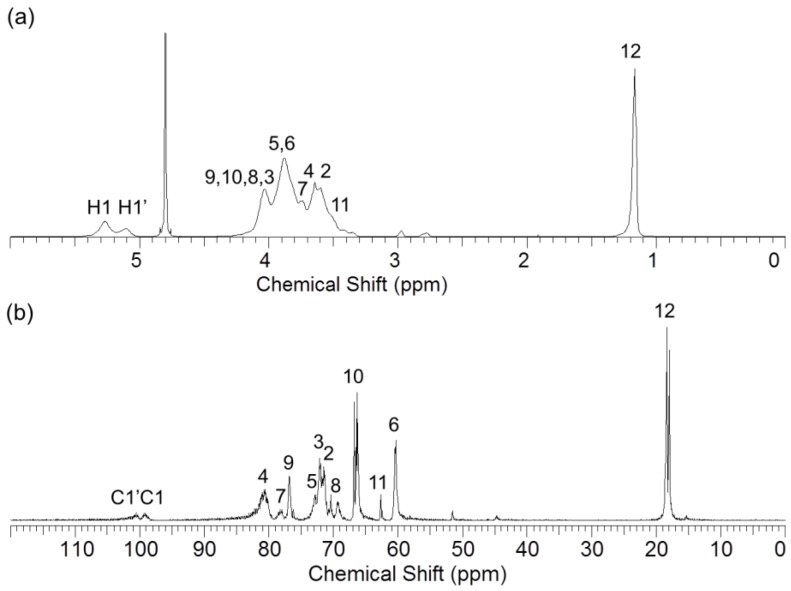
(**a**) ^1^H NMR spectrum and (**b**) ^13^C NMR spectrum of HP-β-CD-ol in D_2_O solvent.

HP-β-CD-ol was synthesized using EP. The synthesized products were separated into oligomers and monomers by size exclusion column chromatography. The separated HP-β-CD-ol was analyzed with MALDI-TOF MS, ^1^H NMR, ^13^C NMR, and HSQC spectroscopy. The chemical structures of EP crosslinked HP-β-CD dimers of oligomers are shown in Figure 1. Since EP was used as a cross-linker, the reaction products have glyceryl bridges and glycerol tails of various lengths. The mass range of HP-β-CD-ol can be observed in the MALDI-TOF mass spectrum (Figure 2). Although the oligomer contains the HP-β-CD dimer, trimer, and tetramer, the dimer was the major product. The average molecular weight (*M*n) of the oligomer was 4202 Da. The structure of HP-β-CD-ol was also analyzed using ^1^H NMR and ^13^C NMR spectroscopy (Figure 3). The –CH and –CH_2_ protons of HP-β-CD appeared in the region of 3.5–4.5 ppm, except for the H1 proton. The –CH_3_ proton was observed at δ 1.12 ppm. The –CH_2_–CHOH–CH_2_– protons of the linker appeared at δ 4.09 (H9), 4.04 (H10), and 3.51 (H11) ppm, respectively. In ^13^C NMR spectrum, the methyl of the hydroxypropyl group signal was assigned at 18.3 ppm, while the glyceryl group protons were assigned at 76.8 (H9), 66.7 (H10) and 62.7 (H11) ppm. These results indicated that the EP residue was successfully substituted at HP-β-CD.

### 3.2. Solubility Enhancement Test

**Figure 4:**
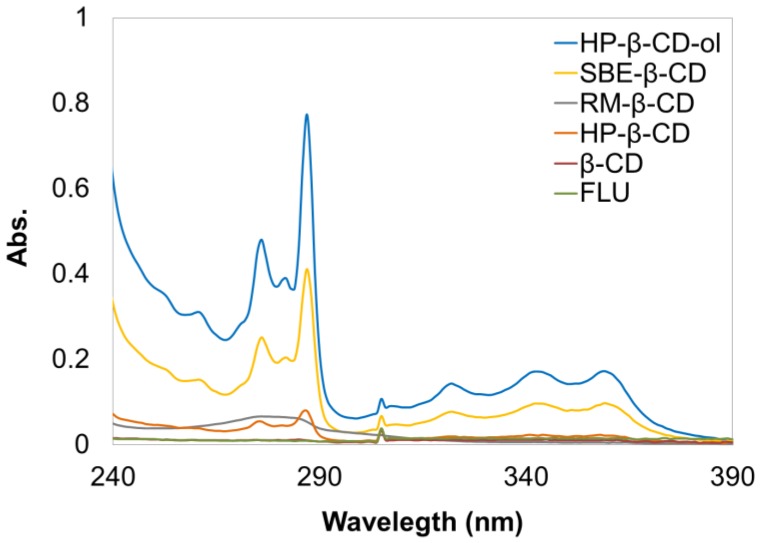
UV-spectra of FT in the presence of 1 mM β-CD, HP-β-CD, RM-β-CD, SBE-β-CD, and HP-β-CD-ol in water at 25 °C.

Solubility enhancement test was conducted as described in the experimental Section 2.4. To compare the FT solubilization capacity of different types of β-CD derivatives, the same amount (200 µg) of FT was equilibrated in the presence of 1 mM synthetic HP-β-CD-ol and other β-CD derivatives (SBE-β-CD, RM-β-CD, HP-β-CD, and β-CD) aqueous solution (1 mL), respectively. Figure 4 shows the UV absorption spectrum of the dissolved FT. The result indicated that HP-β-CD-ol highly enhanced the aqueous solubility of FT compared to the other β-CD derivative monomers. The amount of solubility enhancement was in the order of HP-β-CD-ol > SBE-β-CD > HP-β-CD ≥ RM-β-CD > β-CD > pure FT.

### 3.3. Phase Solubility Tests

**Figure 5:**
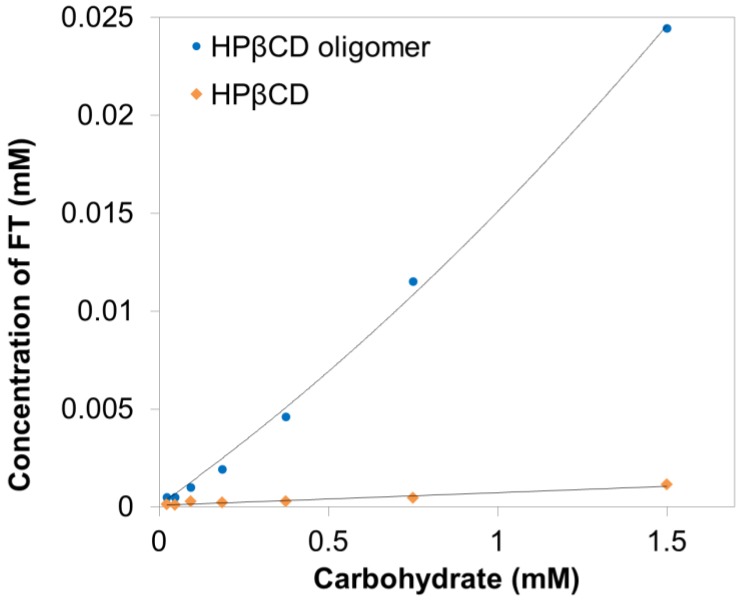
Phase solubility diagrams of FT as a function of HP-β-CD (♦) and HP-β-CD-ol (●) in water at 25 °C.

Phase solubility tests are a commonly used method to determine the effect of cyclodextrin complexation on the solubility of guest molecules (Figure 5). Phase solubility studies of FT were carried out with HP-β-CD and HP-β-CD-ol using the Higuchi and Connors method [22]. The solubility of FT linearly increased as a function of the HP-β-CD concentration. The diagram of HP-β-CD shows A_L_-type slope. It indicates that the FT/HP-β-CD complexes were 1:1 molecular complexes. On the other hand, HP-β-CD-ol shows the A_p_ type slope in the diagram, which suggests that FT/HP-β-CD-ol complexes co-exist at 1:1 and 2:1. The binding constants of FT with HP-β-CD and HP-β-CD-ol were obtained by Equations (1) and (2), while the solubility efficiency was obtained using Equation (4). The stability constant (*K*c) and solubilization efficiency (SE) thus calculated are listed in Table 1. The stability constant (*K*c) is an indication of the binding strength between FT and CDs. HP-β-CD had a *K*c of 6004 M^−1^. The FT/HP-β-CD complexes forming both, 1:1 and 2:1 complexes, had *K*c values of 159,504 and 2195 M^−1^, respectively. Considering the binding constants, the 1:1 complex is more dominant and stable than the 2:1 complex. The intrinsic water solubility of FT is also known as 1.28 μM at 25 °C [23]. The solubility of FT in the presence of 1.5 mM HP-β-CD was 8.3 times higher than that of the original FT. The solubility of FT increased up to 178.3 times by complexation with 1.5 mM HP-β-CD-ol. Thus, the oligomerization of HP-β-CD was highly effective for the solubilization of FT via inclusion complexes. HP-β-CD-ol can provide additional recognition sites due to the glyceryl bridges, glycerol tails, and additional cavity. In particular, the epichlorohydrin reacted HP-β-CD-ol has the hyper-branched structure made of different lengthened tails or only 2-hydroxypropyl ether segments, which would be favorable for the superior supramolecular complexation.

### 3.4. FT-IR Spectroscopic Analysis

**Figure 6:**
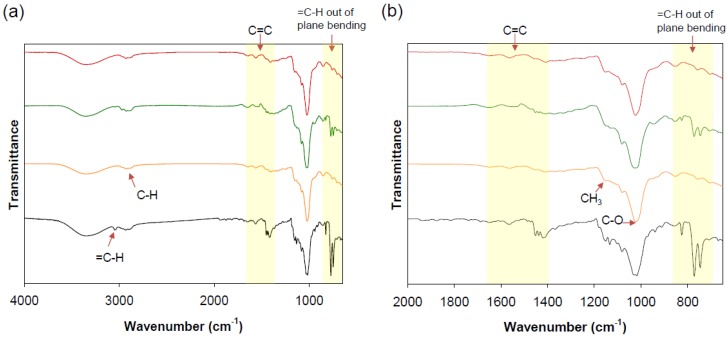
FT-IR spectra FT (black), HP-β-CD-ol (orange), PM (green), and IC (red). (**a**) Spectra were acquired between 4000 and 650 cm^−1^; (**b**) Spectra were acquired between 1700 and 650 cm^−1^.

The physico-chemical properties of FT changed after the inclusion complexation. FT-IR spectroscopy, DSC, and SEM were analyzed to confirm the successful formation of the inclusion complexes. The vibrational changes of FT/HP-β-CD-ol were monitored by FT-IR spectroscopy. The chemical interaction between the two molecules shows a recognizable change in intensity, shape, and peak shift in the infrared spectrum of the composite [24]. The PM of FT/HP-β-CD oligomer was used for comparison with the FT/HP-β-CD oligomer IC. The FT-IR spectra of FT (black), HP-β-CD-ol (green), PM (blue), and IC (red) are shown in Figure 6. The FT IR spectrum showed the characteristic absorbance from 1650 to 1420 cm^−1^ corresponding to the C=C aromatic stretches. The =C–H aromatic out-of-plane banding occurs at from 900 to 690 cm^−1^. The peak at 3354 cm^−1^ corresponds to the –OH vibration in HP-β-CD-ol. The C-H stretching vibration bands appeared at 2930 cm^−1^. The CH_2_ stretching vibration and CH_3_ stretching vibration were detected at 1408 cm^−1^ and 1367 cm^−1^ respectively. Meanwhile, the C–O stretching vibration and C–O–C vibration were shown at 1022 and 1080 cm^−1^. The FT-IR spectrum of the PM contains absorption peaks with a reduced intensity at the same sites as the pure FT. Furthermore, HP-β-CD-ol peaks are also seen at the same positions. This can be due to the simple addition of FT and HP-β-CD-ol. However, the FT-specific absorption peaks disappear in the complex product. This indicates that the physical properties of FT were modified through intensive molecular interactions within the inclusion complex. This confirms the complex formation of FT and HP-β-CD-ol. All the observed IR absorption peaks are presented in Table 2.

### 3.5. DSC Analysis

**Figure 7:**
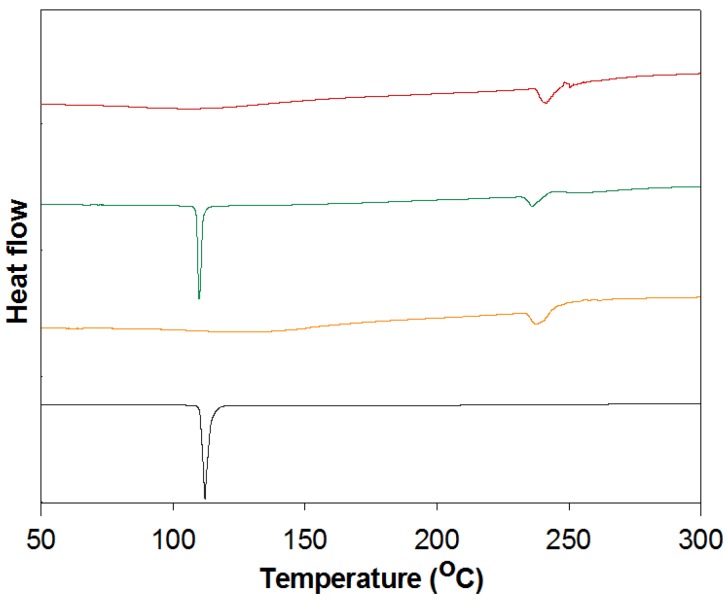
DSC curves of FT (black), HP-β-CD-ol (orange), PM (green), and IC (red).

DSC analysis characterizes the interaction between the host and guest molecules in a solid state [25]. Figure 7 shows the DSC thermogram of FT, HP-β-CD-ol, PM, and IC. FT has a sharp endothermic peak at 112.1 °C, which was its melting point. HP-β-CD-ol has an endothermic peak at 237.9 °C. Similar endothermic peaks of FT and HP-β-CD oligomer were also detected in the DSC curve of PM. However, in the DSC curve of IC, the endothermic peak of FT disappears, while that of CD is slightly shifted to 241.4 °C. These results suggest that the formation of complexes containing FT and HP-β-CD-ol induces a change in the crystal state of FT.

### 3.6. SEM Analysis

**Figure 8:**
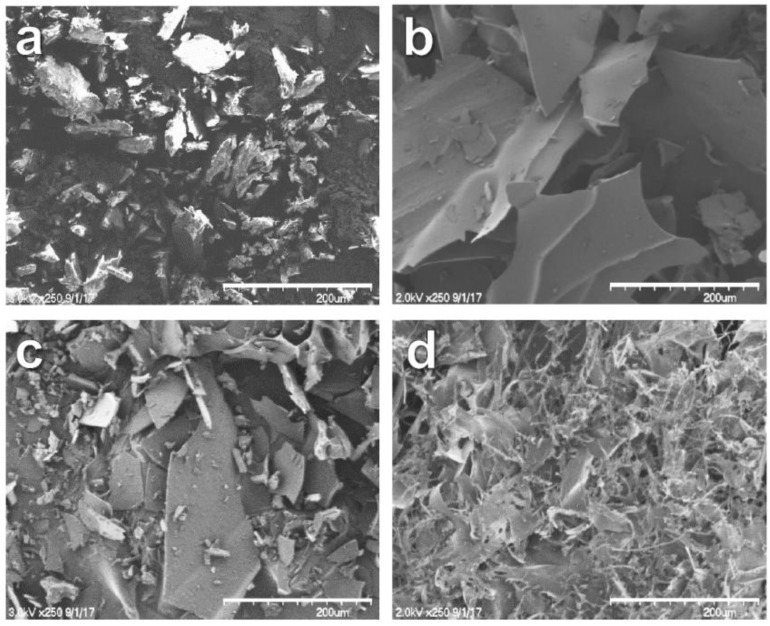
SEM images of (**a**) FT; (**b**) HP-β-CD-ol; (**c**) PM; and (**d**) IC. (×250 magnification, bar = 200 μm).

SEM is used to characterize the morphology changes of inclusion complexes [26,27]. The SEM images of FT, HP-β-CD-ol, PM, and IC are shown in Figure 8. A crystalline particle shape appeared on FT, while a thin plate shape appeared on HP-β-CD. PM shows a mixed shape with FT and HP-β-CD-ol. However, IC shows an amorphous form, unlike the FT and HP-β-CD oligomers. This type of change may be due to the improved dispersion of FT using HP-β-CD-ol. This result confirms the formation of FT/HP-β-CD-ol inclusion complexes.

### 3.7. NOESY Spectroscopy of the FT/HP-β-CD Oligomer Complexes

**Figure 9:**
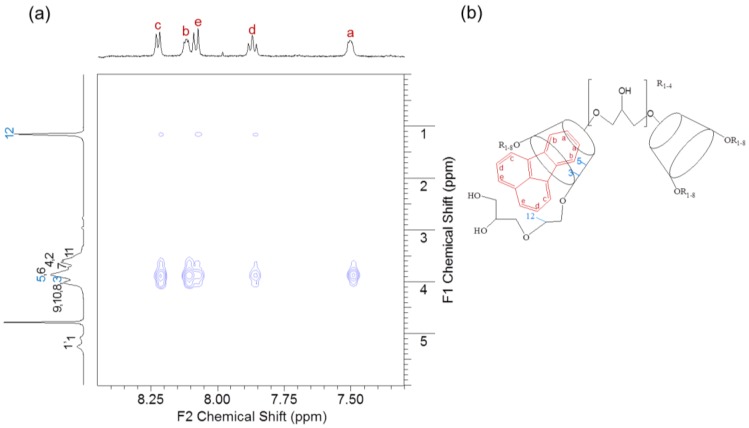
(**a**) NOESY spectrum of FT/HP-β-CD-ol IC in D_2_O solvent with a mixing time of 500 ms; (**b**) Proposed model of FT/HP-β-CD-ol IC.

The 2D NMR spectroscopy is valuable for evaluating the non-covalent interactions at a molecular level in inclusion complex [28]. The NOE peaks appear when two protons are closely located within a distance of 5 Å [29]. The NOESY spectrum of FT/HP-β-CD-ol inclusion complex is shown in Figure 9a. The NOE peaks provide complex model information, from the correlation between the FT and HP-β-CD-ol. Clear cross-peaks were observed between the H-c/H-b/H-e/H-d/H-a protons of FT at 8.23, 8.22/8.12/8.09, 8.07/7.88, 7.87, and 7.85/7.50 ppm, and the inner cavity H-3/H-5 protons of HP-β-CD-ol at 4.04/3.88 ppm. H-b and H-a protons strongly interacted with the H-5 protons of HP-β-CD. These results indicate that the benzene ring of FT was located in or near the inner cavity of the HP-β-CD-ol. Furthermore, the inner cavity size (6–6.5 Å) of β-CD is suitable for the benzene size (5 Å) [30]. H-c/H-d/H-e protons of FT were interestingly correlated with the H-12 proton of HP-β-CD-ol at 1.17 ppm. However, there are no crosses peaks between H-b/H-a of FT and H-12 of HP-β-CD-ol. These results suggest that the naphthalene ring of FT interacts with the hydroxylpropyl moieties of HP-β-CD-ol. The epichlorohydrin hydroxypropyl moiety might assist the FT in entering and immobilizing the inner cavity of the β-CD.

Based on these analyses, we deduced that the benzene ring of FT merged into the HP-β-CD-ol cavity from the wide side, similar to the results of the 1:1 and 1:2 stoichiometry suggested by the phase solubility study. The expected model of FT/HP-β-CD-ol IC is illustrated in Figure 9b. A 1:1 inclusion complex is the dominant type, where a single guest molecule is complexed with a single cyclodextrin.

## 4. Conclusions

FT is produced by the incomplete combustion of organic matter. Its removal from the environment is extremely difficult due to its intrinsic hydrophobic nature. Here, we aimed to solubilize the PAHs using complexation technology in water. The aqueous solubility of FT was successfully enhanced by complexation with the synthesized Epichlorohydrin HP-β-CD-ol. The formation of complexes was analyzed using FT-IR, DSC, FE-SEM, and NMR spectroscopy. On the basis of these results, HP-β-CD-ol is suggested as a potential material for the development of PAH removal systems.

## Figures and Tables

**Table 1:** Binding constants and solubility efficiencies of FT complexes with HP-β-CD-ol.

	Apparent stability constants, *K*_m:n_ (M^−1^)		Solubilization efficiency (SE)
	*K*_(1:1)_	*K*_(1:2)_	
HP-β-CD	6004		8.3
HP-β-CD-ol	159,504	2195	178.3

**Table 2:** FT-IR absorption bands for FT, HP-β-CD-ol, PM, and IC.

	FT	HP-β-CD oligomer	Physical mixture (PM)	Inclusion complex (IC)
O–H		3354	3356	3356
C–H	2931	2930	2926	2930
CH_2_		1408	1409	1408
CH_3_		1367	1372	1366
C–O–C		1080	1081	1080
C–O		1022	1027	1024
≡C–H	3348			
=C–H	3050		3050	
C=C	1650 1562 1474 1452 1438 1420		1650 1561 1452 1438	
